# The Effect of the Preoperative Fasting Regimen on the Incidence of Gastro-Oesophageal Reflux in 90 Dogs

**DOI:** 10.3390/ani12010064

**Published:** 2021-12-29

**Authors:** Paraskevi Tsompanidou, Joris H. Robben, Ioannis Savvas, Tilemahos Anagnostou, Nikitas N. Prassinos, George M. Kazakos

**Affiliations:** 1 Anaesthesiology and Intensive Care Unit, School of Veterinary Medicine, Aristotle University of Thessaloniki, 54627 Thessaloniki, Greece; isavas@vet.auth.gr (I.S.); tanagnos@vet.auth.gr (T.A.); gkdvm@vet.auth.gr (G.M.K.); 2 Section of Emergency and Intensive Care Medicine, Department of Clinical Sciences, Utrecht University, 3584 CM Utrecht, The Netherlands; j.h.robben@uu.nl; 3 Surgery and Obstetrics Unit, School of Veterinary Medicine, Aristotle University of Thessaloniki, 54627 Thessaloniki, Greece; ngreen@vet.auth.gr

**Keywords:** gastro-oesophageal, reflux, GOR, dog, fasting, anaesthesia

## Abstract

**Simple Summary:**

Gastro-oesophageal reflux (GOR), a potential risk during anaesthesia, happens when the stomach contents move up into the oesophagus. The refluxed contents can damage the lining of the oesophagus or the respiratory tract. For many years it was believed that an increase in the stomach contents’ volume increases the risk of GOR. However, more recent studies have demonstrated this to be incorrect. The objective of this study was to compare the effects of three different pre-anaesthetic fasting regimens on the frequency of GOR in dogs under anaesthesia. Ninety dogs undergoing non-abdominal and non-thoracic elective surgery were included in the study and equally allocated to three groups. The results of this study suggest that the administration of a meal 3 h before anaesthesia does not have any beneficial effect in the reduction of GOR incidence in dogs compared to the administration of a meal 12 h before anaesthesia.

**Abstract:**

This study aimed to investigate the effect of three different preoperative fasting regimens on the incidence of gastro-oesophageal reflux (GOR) in dogs under general anaesthesia. Ninety dogs undergoing non-abdominal and non-thoracic elective surgery were included in the study and equally allocated to three groups. Dogs received canned food providing half the daily resting energy requirements (RER) 3 h prior to premedication (group 3H), a quarter of the daily RER 3 h before premedication (group 3Q), and half the daily RER 12 h before premedication (group 12H). The animals were premedicated with acepromazine and pethidine, anaesthesia was induced with propofol and maintained with isoflurane vaporised in oxygen. Oesophageal pH was monitored throughout anaesthesia. Demographic and surgery-related parameters were not different among groups. The incidence of GOR was 11/30 in group 3H (36.7%), 9/30 in group 3Q (30.0%) and 5/30 in group 12H (16.7%), which was not statistically different (*p* = 0.262). Reduction of the amount of the preoperative meal from half to a quarter of the daily RER did not reduce the incidence of GOR but resulted in a lower oesophageal pH (*p* = 0.003). The results of this study suggest that the administration of a meal 3 h before anaesthesia does not have any beneficial effect in the reduction of GOR incidence in dogs compared to the administration of a meal 12 h before anaesthesia.

## 1. Introduction

It is generally believed that prolonged preoperative fasting decreases the risk of gastro-oesophageal reflux (GOR) during anaesthesia. However, this belief is largely based on dogma rather than scientific evidence [1]. In human medicine, the “nil per os” from midnight policy was thought to be mandatory, in order to reduce the volume and acidity of gastric contents, and consequently reduce the risk of regurgitation and aspiration pneumonia in patients undergoing surgery [2,3]. However, the concept that a prolonged fasting period before anaesthesia produces an empty stomach has been demonstrated to be incorrect. In fact, the consumption of solid food shortly before surgery (2–4 h in humans and 3 h in dogs), did not result in statistically significant greater gastric content volumes, compared to an overnight fast [4,5]. Furthermore, there is growing awareness in human medicine that in addition to surgical trauma, prolonged fasting may have detrimental effects on the development of the stress response [6]. The stress response to surgery is characterised by a series of neuroendocrine and inflammatory processes [7], which lead to an increase in whole body catabolism [8]. Reduction of the stress response may improve the patients’ recovery and decrease the risk of organ injury and complications [9]. A similar effect of prolonged fasting may occur in dogs.

Based on the available data in humans, various professional medical organisations currently recommend a short period of fasting prior to the induction of anaesthesia in healthy patients undergoing elective procedures. In order to provide sufficient safety margins, it has been recommended that the fasting period for solids should not be more than 6 h, while oral intake of clear fluids and carbohydrate-rich drinks is permitted up to 2 h prior to the induction of anaesthesia [10,11,12]. In dogs, overnight fasting is recommended by some authors [13], while others suggest that food should not be withheld for more than 6 h [14,15]. Free access to water is usually recommended until premedication [14]. Although a number of studies have been carried out to investigate the effect of preoperative fasting on the incidence of GOR [16,17,18] and on the residual gastric content in dogs undergoing general anaesthesia [4], there is not enough unequivocal data to produce clinical guidelines in dogs.

The objective of this study was to compare the effect of three different preoperative fasting regimens on the incidence of GOR in dogs undergoing non-abdominal and non-thoracic elective surgery. A reduction in the preoperative food withholding period and in the amount of food before anaesthesia was expected to result in a lower incidence of GOR. More precisely, a 3 h preoperative fasting was expected to result in a lower incidence of GOR episodes as compared to a 12 h fasting. The reduction of the ingested meal from half to a quarter of the daily resting energy requirements (RER), three hours before the premedication, was also expected to result in a lower incidence of GOR. Additionally, a 12 h fasting was expected to result in lower pH values compared to a 3 h fasting. 

## 2. Materials and Methods

### 2.1. Cases and Pre-Anaesthetic Management

The current work was approved by the Institution’s Ethical Committee (protocol ID 19/5-6-2012). Ninety client-owned dogs admitted for elective surgeries (non-emergency surgeries that could be planned in advance) were included in this study, after the informed written consent of their owners. Inclusion criteria were non-abdominal and non-thoracic surgery. Eligible for inclusion were dogs between 1 and 10 years old, with a body weight (BW) over 5 kg, and an American Society of Anaesthesiologists (ASA) physical status I or II. Exclusion criteria were history of active or probable gastrointestinal disease, as well as the administration of medication, during the last five days, that could influence the function and motility of the gastrointestinal tract (i.e., antacids, histamine type-2 receptor antagonists, proton pump inhibitors, metoclopramide and maropitant citrate). The health status assessment of the dogs was based on physical examination. Complete blood count and serum biochemical profile were performed in all dogs over five years of age, and animals with abnormal findings were excluded from the study.

All dogs were hospitalised for at least 24 h before surgery. All animals were deprived of food for 12 h before the administration of the test meal and had free access to water up to 3 h before the administration of premedication. The dogs were allocated into three groups in a sequential, non-randomised manner. If a dog had to be excluded from the study, then the next dog which would meet the inclusion and exclusion criteria was allocated in the same group/strata of the excluded dog. The dogs of the first group (group 3H) received canned food (Apollo; NutriPet, Kilkis, Greece) providing half the RER 3 h before the administration of the premedication. The second group (group 3Q) received canned food at a quarter of the RER 3 h before premedication. The animals of the third group (control group, group 12H) received canned food at a quantity equal to half the RER 12 h prior to premedication. The daily RERs were calculated based on the following formula: E = 70 × (BW [kg])^0.75^. The dry matter composition of the food offered to the dogs was the following: protein 37.8%, fat 29.7%, fibre 2.7%, others 29.7%.

### 2.2. Anaesthetic Management

All dogs were premedicated with 0.02 mg/kg BW of acepromazine maleate (Acepromazine; Alfasan, Woerden, The Netherlands) and 3 mg/kg BW of pethidine (Petidina cloridrato; Molteni, Florence, Italy) mixed in the same syringe, intramuscularly at the femoral biceps muscle. After the administration of the pre-anaesthetic medication, the dogs were placed in a quiet cage and left undisturbed for 30 min. Subsequently, an intravenous catheter was placed in a cephalic vein and the infusion of a Lactated Ringer’s solution (LR’s; Vioser, Trikala, Greece) at 10 mL/kg/h BW was initiated. Anaesthesia was induced with propofol (Propofol MCT/LCT; Fresenius Kabi Hellas, Athens, Greece), at repeated doses of 1 mg/kg BW until adequate suppression of the laryngeal reflexes allowed for endotracheal intubation. The total dose of propofol administered to each animal was recorded. Anaesthesia was maintained with isoflurane (Isoflurane-Vet; Merial, Milano, Italy) vaporised in oxygen through a semi-closed circle system or a non-rebreathing system for patients weighing less than 8 kg. End-tidal isoflurane concentration was adjusted based on the patient’s response to surgical stimulation, in terms of arterial blood pressure, heart and respiratory rate. For intraoperative analgesia, a fentanyl citrate (Fentanyl; Janssen- Cilag, Athens, Greece) constant rate infusion at 0.03 μg/kg/min BW was administered throughout the surgery. Carprofen (Rimadyl; Phizer Hellas, Athens, Greece), at 2 mg/kg BW IV, was administrated after intubation and was continued every 12 h after the initial administration. If additional analgesia had been required during the surgery, an adjustment of the fentanyl CRI dose would have been done and/or supplementary boluses of fentanyl would have been administered. These animals would be excluded from the study. During anaesthesia, heart and respiratory rate, electrocardiography, indirect oscillometric systolic, diastolic and mean arterial blood pressure, end-tidal CO_2_ (Datex-Ohmeda S/5, General Electric Healthcare, Helsinki, Finland) and end-tidal isoflurane concentration (Capnomac Ultima, Datex-Engstrom, Helsinki, Finland) were continuously monitored. The animals were allowed to breathe spontaneously. Intermittent positive pressure ventilation (IPPV) was started whenever the end-tidal CO_2_ exceeded 55 mmHg, and these dogs were subsequently excluded from the study. Warm-water blankets were used to prevent hypothermia and rectal temperature was recorded upon initiation and at the end of surgery.

### 2.3. PH Measurement

After intubation, a pH sensing probe connected to a portable pH meter (pH electrode 52-00; pH meter 507, Crison Instruments SA, Barcelona, Spain) was introduced into the oesophagus for the pH measurements, and detection of GOR. The electrode was calibrated before each use with buffer solutions (pH 4 and 7). The correct placement of the probe into the oesophagus was ensured by externally measuring the distance between the incisor teeth on the lower jaw and the cranial margin of the tenth rib, through the angle of the mandible, and subsequently subtracting 5 cm from the premeasured length [19]. In all cases, the probe placement was performed by the same investigator. The oesophageal pH was monitored constantly and recorded every 5 min throughout anaesthesia. Gastro-oesophageal reflux was considered to have occurred if a pH < 4 or >7.5 was recorded [20].

### 2.4. Postoperative Management of GOR Cases

A seven-day treatment with ranitidine (Zantac; GlaxoSmithKline Hellas, Athens, Greece, 2 mg/kg BW, PO, BID), metoclopramide (Primperan; Sanofi-aventis Hellas, Athens, Greece, 0.3 mg/kg BW, PO, BID) and sucralfate (Peptonorm; Uni-Pharma, Athens, Greece, 30 mg/kg BW, QID, PO) was recommended for all the dogs that experienced GOR. The owners of these animals were informed about the possible complications, such as oesophagitis and oesophageal stricture, and were instructed to contact the clinic if their dogs showed any sign of oesophageal dysfunction, such as unexplained anorexia, regurgitation, vomiting or hypersalivation.

### 2.5. Statistical Analysis

An a priori power analysis revealed that in order to achieve 0.8 power of the statistical analysis, a total sample size of 90, with an effect size of 0.3, was needed. The design of the study was three blocks of equal size, with stratification (two strata) in each block. All groups were stratified by type of surgery (soft tissue (Soft group) or orthopaedic (Ortho group)). This was decided in an effort to allocate the same number of different types of surgery in each group.

Qualitative variables (incidence of GOR among groups, and gender of the dogs) were compared using the chi-squared test of association. A chi-square test was also used to evaluate any effect of the recumbency and the type of surgery on the incidence of GOR. The normality assumption of continuous variables was evaluated with the Shapiro–Wilk test of normality. Variables were found to follow a non-normal distribution; thus, non-parametric statistical tests were used. Logistic regression analysis was used to evaluate any effect of quantitative variables (gender, age, BW, duration of anaesthesia, duration of surgery, dose of propofol) on the incidence of GOR. The Kruskal–Wallis test was used to reveal any differences in the pH during GOR episodes, the time of onset of GOR, and the duration of GOR among the groups. It was also used to evaluate for any differences among the groups for age, BW, dose of propofol, duration of anaesthesia and duration of surgery. Additionally, it was used to evaluate for any effect of the type of surgery on the onset of GOR and the acidity of the refluxate. The Mann–Whitney test was used for the pairwise comparisons. Statistical significance was set to 0.05, except when the Mann–Whitney test was used i.e., 0.013 after the Bonferroni correction was applied. For all statistical tests the IBM SPSS Statistics for Mac, V.24.0 computer software was used (IBM, Armonk, New York, NY, USA).

## 3. Results

All groups were not statistically different for gender (*p* = 0.798), age (*p* = 0.124) and BW (*p* = 0.900, Table 1). Statistical analysis did not demonstrate any difference among the groups concerning the duration of anaesthesia (*p* = 0.922) and surgery (*p* = 0.726, Table 1). All groups were similar with regard to the frequency of the surgical procedures. Of the 90 patients, 33 underwent soft tissue surgery (11 in each group), while the remaining 57 patients underwent orthopaedic surgery (19 in each group). Four dogs were excluded from the study and subsequently replaced. One dog was excluded because it presented diarrhoea the night before the surgery, two because they refused to eat the control meal, and one because it presented hypercapnia during the surgery.

No statistical difference was found between the three groups regarding the incidence of GOR (*p* = 0.262). The number of GOR cases in each group is shown in Table 2. When the incidence of GOR was combined for the 3 h fasting groups, 20 out of 60 dogs (33.3%) experienced a GOR episode. Eleven out of 30 dogs (36.7%) in group 3H, 9/30 (30.0%) in group 3Q and 5/30 (16.7%) in group 12H experienced GOR (*p* = 0.262, Table 2). In all cases, the dogs experienced one episode of GOR. During the GOR episodes, the median pH in group 3H was 1.5 (1.1 to 3.7), in group 3Q 1.0 (0.6 to 1.9) and in group 12H 1.4 (0.6 to 2.9). The incidence of GOR did not differ significantly between the two genders (*p* = 0.235). No effect of age (*p* = 1.000) or BW (*p* = 1.000) of the animals was detected on the incidence of GOR. The dose of propofol per kilogram of BW administered to each animal, the duration of anaesthesia and the duration of surgery did not have any effect on the incidence of GOR (*p* = 1.000 for all comparisons). In 17 out of the 25 cases of GOR, the pH value was lower than 4 within 25 min of the induction of anaesthesia and prior to the initiation of surgery. In 7 of these cases, a pH value lower than 4 was detected at the time of insertion of the pH measuring probe, suggesting that GOR took place during the induction of anaesthesia or before that. No statistical difference was found in the time of onset of GOR (*p* = 0.525) or the duration of GOR (*p* = 0.636) between the three groups (Table 2).

On all occasions of GOR, the refluxate was acidic. The pairwise comparisons revealed that the pH, during the episodes of GOR, was significantly lower in group 3Q when compared with group 3H (*p* = 0.003). No significant differences in the pH values were found between group 3H and group 12H (*p* = 0.829) or group 3Q and group 12H (*p* = 0.588, Table 2).

The recumbency of the dogs was not identified as a risk factor for the development of GOR (*p* = 0.521). A total of 13 out of 39 dogs (33.3%) were placed in lateral, 6/29 (20.6%) in dorsal, and 6/22 (27.2%) in sternal recumbency. Seven out of 33 (21.2%) dogs undergoing soft tissue surgery and 18/57 (31.6%) undergoing orthopaedic surgery experienced GOR (*p* = 0.337). The type of surgical procedure was not found to affect the time of onset of GOR (*p* = 0.804) or the acidity of the refluxate (*p* = 0.688, Table 2). No difference was found in the duration of the GOR episodes between the dogs undergoing orthopaedic and soft tissue surgeries (*p* = 0.043). The duration of anaesthesia and surgery in the dogs undergoing soft tissue surgery was 115 (60–170) and 65 (35–120) min respectively, while for the dogs undergoing orthopaedic surgeries was 165 (105–240) and 120 (75–190) min.

Regurgitation was observed in one animal of group 3H, placed in lateral recumbency undergoing orthopaedic surgery. At the end of the surgery, the gastric contents were suctioned, and lavage of the oesophageal lumen was performed using tap water. The lavage was repeated several times until the suctioned fluid was clear. 

No episodes of vomiting, salivation or oesophageal dysfunction were observed in any of the animals during their hospitalisation in the postoperative period. Additionally, no cases of oesophagitis or oesophageal stricture were reported after discharge.

## 4. Discussion

In the present study, the choice of the quantity of the preoperative meal was based on the results reported by Savvas et al. [4] that suggested a meal containing half the RER 3 h prior to the induction of anaesthesia does not increase the stomach volume significantly compared to a 10 h fasting period. However, in the same study, the mean gastric volume of the dogs fed canned food 3 h before the induction of anaesthesia was almost three times the volume of those fed the same type and amount of food 10 h prior to anaesthesia [3]. In humans, there was no increase in the gastric volume after a light breakfast of tea and buttered toast consumed 2–4 h before surgery. Despite that, in the aforementioned study, the presence of residual solids in the stomach at the time of induction could not be ruled out [5]. Based on these findings, and in an attempt to further reduce the residual gastric volume, a third treatment group was included in our study, with dogs fed a meal containing a quarter of RER 3 h before anaesthesia (group 3Q).

The time periods of food withholding in the present study were 12 h, in order to simulate the overnight fasting commonly suggested in veterinary practice, and 3 h, which according to previous studies [16,18] could be of benefit in reducing the incidence of GOR in dogs during anaesthesia. Although not statistically significant, the 12 h group presented the lowest incidence of GOR (16.7%). The occurrence of GOR observed in our 12 h group is in accordance with other studies which reported an incidence of GOR ranging from 13.3% to 20% in their 12 to 18 h groups [16,18]. 

Reported incidences of GOR in the veterinary literature vary widely. With a fasting period of 3 h, the reported GOR episodes have been as low as 0 to 5% and as high as 61% [16,17,18,21]. In our study, the combined incidence of GOR in the two 3 h fasting groups together was 33.3% (20/60 dogs). The amount and composition of the meals in combination with the anaesthetic protocol in the aforementioned studies may have attributed to the large variation in results. Some studies did not report the composition of the meals used [16]. A high incidence of GOR was observed in a study that used a canned recovery diet [17]. In the present study, as in the study of Savvas et al. [18], a commercially available canned food was used, with a lower protein and fat content compared to recovery diets. However, the effect of food composition on the incidence of GOR in dogs has not been extensively studied. Additionally, no definitive conclusions have been reached concerning the effect of the diet composition on the occurrence of GOR in humans [22,23,24]. High protein and fat diets play a role in gastric emptying and may, possibly, have a consequence on the incidence of GOR in dogs.

The anaesthetic protocol is another factor that could influence the barrier function of the lower oesophageal sphincter (LOS) and, as a result, the incidence of GOR in dogs. In the only study that is similar to the currently presented work, in regard to the fasting periods and the type of food [18], propionyl-promazine was used as premedication whereas in the current study acepromazine was used. Both belong to the same drug group, but the relaxing effect of acepromazine may be more pronounced than the effect of propionyl-promazine on LOS [25,26]. Furthermore, the use of full μ-agonist opioids in premedication has been shown to increase the incidence of GOR and regurgitation in dogs [26,27]. The combined use of acepromazine with opioids (pethidine and fentanyl) as part of the anaesthetic protocol in the present study might have contributed to the higher incidence of GOR compared to the study of Savvas et al., 2016 [18]. In the present study, a low fentanyl dose was used as a constant rate infusion intraoperatively. This was made in an attempt to preserve eucapnia and avoid the use of IPPV, which would have led to exclusion of these animals from the study. Even though no dogs required additional analgesia during our study, if such cases had risen, we would have reconsidered our analgesic plan. 

Propofol may have contributed to the higher incidence of GOR observed in our study compared to other studies that used thiopental as an induction agent [18,21]. Propofol, compared to thiopental, causes a more profound relaxation of the LOS [28], and a significantly higher incidence of GOR during anaesthesia in dogs [29]. As maintenance of anaesthesia with halothane, isoflurane and sevoflurane has been associated with a similar risk for the development of GOR in dogs, their use does not seem to explain the differences reported among the studies [30]. 

Previous studies have reported that prolonged preoperative fasting periods are associated with increased gastric acidity [4,16,17]. Galatos and Raptopoulos (1995a) found that dogs fasted for at least 24 h have a significantly lower gastric pH compared to those fasted for 2–4 h or 12–18 h [16]. However, in that study, no significant difference was found between the 2–4 and 12–18 h groups. This accords with the results of the present study which found no significant difference in the pH between the 3 and 12 h fasting groups. These findings are in contrast with a study in which a 10 h fasting period produced a lower pH than a 3 h period [4]. The authors of the latter study hypothesised that with a longer fasting period food would not be in the stomach anymore to exert its buffering effect on gastric acidity. In humans, the gastric pH increases after a meal due to the buffering effect of the ingested food. The postprandial pH in humans starts from 7 and becomes acidic again in 60–90 min by post-prandial gastric acid secretions. Studies in conscious dogs found that during and following a meal the dog’s gastric pH remains relatively acidic and that, in contrast to humans, there is less buffering effect of food [31,32]. Additionally, lower volumes of food with less buffering capacity have been associated with lower values of gastric pH. More precisely, the gastric pH in dogs fed 10 gr of dry food was significantly lower compared to that in dogs fed 200 gr of the same food [32]. This latter observation is in agreement with our findings where the pH of the refluxed material in group 3Q was significantly lower compared to that of group 3H.

In the present study, the GOR events were acidic on all occasions, suggesting that the refluxate was primarily of gastric origin. This finding is in agreement with previous studies which reported either only acidic episodes of GOR or a low incidence of duodenogastro-oesophageal reflux episodes [16,18,26,30].

In this study, an increase in the duration of anaesthesia and surgery did not increase the incidence of GOR, which corresponds with previous studies [17,33]. Furthermore, no significant differences were observed among the three groups concerning the time of onset and the duration of GOR. Gastro-oesophageal reflux usually occurred shortly after the induction of anaesthesia, which is in agreement with previous reports [16,26,33,34]. Additionally, in agreement with a previous study [35], no relationship was established between the type of surgery and the incidence of GOR. Our research confirms earlier findings that gender [33,35], age [26,36,37,38] and recumbency [39] of the animals do not predispose for the development of GOR in dogs under anaesthesia.

No signs of oesophagitis, oesophageal stricture or aspiration pneumonia were observed in the postoperative period in any of the dogs that experienced GOR during anaesthesia. However, we relied on the observation of owners and did not perform postoperative endoscopy, and minor oesophageal damage could have easily been missed. Moreover, our medical treatment could have prevented the development of oesophageal complications. Our findings are in agreement with previous studies that have reported no cases of oesophageal dysfunction or aspiration pneumonia in dogs after anaesthesia, despite the high incidence of GOR [21,29,37].

The distribution of dogs in the three groups was not blinded to the main investigator, as the main investigator was responsible for the dog feeding, anaesthesia and oesophageal pH probe placement. Because the data obtained were objective (oesophageal pH) it is not likely that this fact affected the results presented in the current study.

The non-random allocation of the dogs in the three groups does introduce the potential of selection bias. However, the choice of the groups was not based on the investigators’ personal preference for a specific group, nor on the patients’ demographic characteristics, but in the order that these animals were admitted to the clinic. 

Another limitation of the present study is that detection of GOR episodes was based only on oesophageal pH measurement. Oesophageal pH-metry is a sensitive technique in identifying acid reflux episodes, but it cannot reliably detect reflux episodes with a pH > 4 [40]. It is possible that such episodes might have gone unnoticed in our study. Combined impedance and pH monitoring could have allowed the detection of reflux episodes of intermediate pH (4 < pH < 7.5) [41]. As in most of the previous studies investigating the incidence of GOR in dogs, the correct placement of the pH probe was not confirmed with x-rays [16,33,37,38,42], in order to avoid any body position changes or changes in the anaesthetic depth, that could have potentially resulted in an increase in the episodes of GOR. 

## 5. Conclusions

This study did not reveal a difference in the incidence of GOR episodes between dogs under general anaesthesia with different preoperative fasting periods. As a shorter period of fasting could be advantageous in preventing metabolic changes related to fasting, the results of the present study do not oppose the choice for a shorter preoperative fasting period. However, the results of the present study indicate the importance of the amount of food administered before anaesthesia as the reduction of the amount of the preoperative meal from half to a quarter of the RER was related to a higher acidity of the refluxate which could potentially have adverse effects.

## Figures and Tables

**Table 1:** Descriptive statistics of gender, age, body weight (BW), propofol dosage, duration of anaesthesia and surgery of the thirty dogs in each study group.

	Group 3H ^a^	Group 3Q ^b^	Group 12H ^c^
Total number of dogs	30	30	30
Males	18	19	16
Females	12	11	14
Age (years) *	2.5 (1.0–10.0)	3.0 (1.0–8.0)	6.0 (1.0–10.0)
BW (kg) *	21 (5.0–50.0)	23.5 (5.0–39.0)	19.5 (7.5–37.0)
Propofol (mg/kg BW) *	5.0 (4.0–7.5)	5.7 (3.0–8.0)	5.0 (3.0–9.0)
Duration of anaesthesia (mins) *	145 (65–235)	152.5 (60–240)	147 (70–240)
Duration of surgery (mins) *	100.0 (35–180)	112.5 (35–190)	95.0 (35–180)

^a^ dogs fed half of the resting energy requirements (RER) three hours prior to premedication, ^b^ dogs fed a quarter of the RER three hours prior to premedication, ^c^ dogs fed half of the RER 12 h prior to premedication; * values expressed as median (range).

**Table 2:** Descriptive statistics of the episodes of gastro-oesophageal reflux (GOR) in each of the three study groups.

	Group 3H ^a^	Group 3Q ^b^	Group 12H ^c^
Total number of dogs	30	30	30
Cases of GOR (number)	11	9	5
Cases of GOR (%)	36.7	30.0	16.7
Onset of GOR * (mins)	25 (1–60)	5 (1–120)	5 (1–35)
Duration of GOR * (mins)	90 (50–125)	95 (40–235)	90 (10–150)
Minimum pH *	1.5 (1.1–3.7) ^†^	1.0 (0.6–1.9) ^†^	1.4 (0.6–2.9)

^a^ dogs fed half of the resting energy requirements (RER) three hours prior to premedication, ^b^ dogs fed a quarter of the RER three hours prior to premedication, ^c^ dogs fed half of the RER 12 h prior to premedication; * values expressed as median (range); ^†^ values significantly different between the groups (*p* = 0.003).

## Data Availability

The data presented in this study are available on request from the corresponding author.
